# Cruel Treatment of a Lunatic by Her Parents

**Published:** 1887-10-15

**Authors:** 


### CRUEL TREATMENT OF A LUNATIC BY HER PARENTS.

(See Vol. II, p. 322; and Vol. III, p. 17.)

THE mother of this patient writes to us again as follows:—"My daughter has become chargeable to the Union as a pauper lunatic, from 17th February 1886, up to this date, but why I cannot tell. It would appear that some one has misinformed you. I thank you most sincerely for your courteous attention. If everyone had been as straightforward as yourself, we should have had our dear child now. I should like to inquire whether parents can be compelled to pay for their child as a pauper, when they have a superior home to offer, in a free country like England? My daughter comes of noble birth, and has never received parochial relief, and I cannot understand why she is called a pauper; yet we have always paid for her, because they threatened to summon us—though I should have let them summon me, because I should have had an opportunity to ask how they could do so, when we were not refusing to maintain our child. We wish to do so in a far better way, by keeping her in her own position. She was put in upon hearsay evidence, consequently no one knew how to fill up the order correctly; in fact, I cannot understand how they could do it legally."

*** Section 68 of the 97th chapter of the 16 and 17 Vict. provides for the admission of the lunatic into an asylum under the circumstances referred to by our correspondent, and it seems beyond doubt that the Act has been complied with. The Act, however states "that nothing herein contained shall be construed to extend, to restrain, or prevent any relation or friend from retaining or taking such lunatic under his own care, if such relation or friend shall satisfy the Justice or Justices before whom such lunatic shall be brought, or the visitors of the asylum in which such lunatic is or is intended to be placed, that such lunatic will be properly taken care of." All that our correspondent has to do, therefore, is to "satisfy the Justices," and this she apparently has not done.

The 69th section of the same Act provides for the making of an order upon the Guardians for the maintenance of the ordinary pauper lunatic, and where the lunatic is sent under the 68th section the case would seem to be brought under the 69th section by the operation of the 118th section. (See Fry's Lunacy Acts, page 414.) It would therefore be the Guardians who would compel the relations to pay.

Of course, if our correspondent could show that the lunatic had been illegally sent to the asylum, and illegally detained there, fresh issues might be involved; but we fear she is unable to show any illegality, consequently her only course is to satisfy the Justices that her daughter will be properly taken care of. We may inform her that any three visiting Justices can discharge any patient, so that she has only to convince three out of perhaps twenty that her case is right.

